# Detergent-like stressor and nutrient in metabolism of *Penicillium chrysogenum*

**DOI:** 10.1080/13102818.2014.901674

**Published:** 2014-05-22

**Authors:** Violeta Jakovljević, Jasmina Milićević, Jelica Stojanović

**Affiliations:** ^a^ Institute for Biology and Ecology, Faculty of Science, University of Kragujevac, Kragujevac, Serbia

**Keywords:** biomass, biodegradation of detergent, enzyme activity, organic acids, pH, redox potential

## Abstract

The influence of detergents on the metabolism of *Penicillium chrysogenum* from two aspects, as a stress factor and potential nutrient, was studied. The fungus was isolated from the river bed Lepenica, Kragujevac, at a place where sewage domestic wastewater discharged into the river. The fungus was grown in a liquid nutrient medium according to Czapek with and without addition of commercial detergent (MERIX, Henkel, Serbia) at a concentration of 0.3% and 0.5%. The biochemical changes of pH, redox potential, free and total organic acids, total dry weight biomass, activity of alkaline and acid invertase and alkaline phosphatase were evaluated from day 3 to day 16 of the fungus growth. At the same time, detergent disappearance in terms of methylene blue active substances in the medium was measured. The detergent at a concentration of 0.5% showed a fungicide effect. In the medium with 0.3% of detergent, there was increased pH and concentration of organic acids, but decreased redox potential and total dry weight biomass. The detergent also showed an inhibitory effect on invertase and phosphatase activity. *P. chrysogenum* decomposed 50.2% of the total detergent concentration for an experimental period of 16 days.

## Introduction

Fungi inhabit different environments and are exposed to many extreme factors that limit their survival. These stress factors are as follows: pH changes, osmotic changes, temperature changes, oxide radicals, nutrient deficits and exposure to chemicals.[1] They can originate either from natural environments or from the host immune system of animals and humans in response to pathogenesis. A defence mechanism that fungi use against these stress factors is based on the structure of the cell wall, which is unique in the living world. It consists of α- and β-glucans, *N*-acetylglucosamine, manoproteins and various other glycoproteins. When fungal cells come into contact with these stress factors, the cells respond with expression of genes directed towards synthesis of proteins which enable the cells to survive adverse conditions.[2] Owing to this fact, filamentous fungi have a potential to grow in the presence of various pollutants, to break them down and utilize the degradation products as an energy and carbon source. Among numerous pollutants, commercial detergents stand out especially because of their wide use.

Detergents are complex mixtures containing surface-active agents (SAA), softeners, oxidizing agents and various enzymes (protease, lipase, amylase and cellulase).[3] Detergent pH is very alkaline and ranges from 9 to 12, which is why they are considered strong alkaline agents. It is known that detergents have harmful effects on the hydrobionts and microorganisms in wastewater. Detergents as pollutants get into the environment through industrial and municipal waste water, pesticide application or deposit of waste activated sludge.[4] Discharging of sewage and SAA in their final form in the sediment, the toxicity to aquatic organisms and bioaccumulation in the food chain, along with emissions of CO_2_, SO_2_ and NO_x_, are some of the main problems today.[5,6] Products with a high content of SAA, such as detergents, inhibit the growth of unicellular algae, impede the process of water purification by creating foam, which makes it difficult to dissolve oxygen necessary for breathing and photosynthesis, and cause eutrophication. The maximal allowable concentration of detergent in wastewater effluents in public sewage is 4 and 0.5 mg L^−1^ in natural recipients, according to legislative acts.[7] Identification of fungal species which are resistant to these concentrations of detergents has a great practical application in bioremediation.

*Penicillium chrysogenum* is a ubiquitous soil filamentous fungus, but it can also be present in aquatic environments. This species is industrially used for the production of a number of secondary metabolites, including penicillin, for the treatment of wastewater from pulp mills, for production of enzymes, etc.[8, 9] Some *Penicillium* species are promising candidate bioremediation agents because of their ability to break down different xenobiotic compounds.[10]

Little, however, is known about the effects of commercial detergents as stress factors on the growth of *P. chrysogenum* and its potential to break down detergent and use the degradation products for its metabolism. This study focused on some new aspects of fungal biochemical responses to high concentrations of commercial detergent (several thousand-fold higher than those allowable and lethal for most living beings) and fungal detergent-degrading ability at the same time. The fungal isolate was obtained from municipal wastewater rich in detergent, which is why we assumed that the fungus may have the ability to degrade different pollutants in the wastewater, including detergent. Detergent is a complex pollutant in nature and under standard conditions can be degraded by microorganisms but at a very low rate. Due to these facts, the isolation and investigation of different detergent-degrading microorganism strains is of great practical importance in biotechnology and remediation of natural ecosystems.

## Materials and methods

### Microorganism and culture conditions

The fungal strain was isolated from wastewater samples from the Lepenica River (Kragujevac, Serbia) at a place where domestic sewage wastewater discharges into the river. In late May 2010, a sample of wastewater was collected and transported to the microbiology laboratory. Within 24 h of sample collection, the sample was inoculated onto nutrient malt agar plates with streptomycin, in duplicates. The plates were then incubated at room temperature (28 °C ± 2 °C). Pure culture was obtained by the method of exhausting on poor malt agar plates and potato-dextrose-agar (PDA) plates. Identification of the fungus was done according to systematic key at the Institute of Biology and Ecology, University of Kragujevac. The fungus was maintained on PDA slants grown at 30 °C, stored at (4 ± 0.5) °C, and subcultured monthly in sterile conditions. During the experiment, the fungus was cultivated in sterile Czapek–Dox liquid medium (Table 1).

**Table 1.** Composition of growth medium in 1000 mL distilled water.

		*C* (g L^−1^)					
Growth medium	Denoted	NaNO_3_	K_2_HPO_4_	MgSO_4_·7H_2_O	FeSO_4_·7H_2_O	Sucrose	Detergent
Control	C	3	1	0.5	0.01	30	
C + 0.3% detergent	D3	3	1	0.5	0.01	30	3
C + 0.5% detergent	D5	3	1	0.5	0.01	30	5

Erlenmeyer flasks with growth media were sterilized at 120 °C for 20 min (0.14 MPa). The pH control was adjusted to about 4.70 before sterilization with 0.1 mol L^−1^ HCl.

### Inoculation and sampling

The liquid growth media were stored in Erlenmeyer flasks (200 mL of medium in 250 mL flask). One positive control without detergent with spores, two test flasks with different concentrations of detergent and with spores, and two negative controls with detergent but without spores were used. One millilitre of spore suspension (1×10^4^ CFU mL^−1^) was used for inoculation. Erlenmeyer flasks in three replicates were placed on an electric shaker (Kinetor-m, Ljubljana), thus enabling uniform and constant mixing. All experiments were carried out at room temperature, under alternate light and dark for 16 days. Sampling started three days after inoculation and was repeated every third day until the end of the experiment. Mycelium was removed by filtration through Whatman filter paper No. 1 and mycelial dry weight biomass was determined. The filtrate was harvested by centrifugation at 10 000 r/min for 10 min (4 °C) and the supernatant was used as a crude enzyme extract.

### pH and redox potential

The pH and redox potential were measured by a digital electric pH meter (PHS-3BW Microprocessor pH/mV/Temperature Meter) type of Bante with glass electrode model 65-1.

### Invertase activity

Alkaline invertase activity was assayed in a reaction mixture that consisted of 0.06 mol L^−1^ sucrose, 0.02 mol L^−1^ citrate buffer (pH 8.0) and 300 μL of enzyme solution, in a final volume of 1 mL. The mixture was incubated at 37 ºC for 30 min. The reaction mixture for acid invertase activity contained the same components, except that citrate buffer was replaced with 0.1 mol L^−1^ sodium acetate buffer (pH 4.5). The reaction mixture was incubated at 55 ºC for 30 min. The amount of reducing sugar liberated was determined spectrophotometrically at 410 nm, by the method of Somogyi–Nelson.[11] One unit of invertase activity (IU) was defined as the amount of enzyme which catalyses the production of 1 μmol of glucose per min at 37 ºC.

### Alkaline phosphatase activity

Alkaline phosphatase (ALP) activity was assayed with β-glycerophosphate as a substrate. The reaction mixture contained an equal volume of 0.05 mol L^−1^ glycol buffer (pH 9) with activator (Mg^2+^), substrate and fermentation broth. After incubation at 37 °C for 30 min, the reaction was stopped by adding 10% trichloroacetic acid and was stored for 15 min on ice. NH_4_–molybdate solution was used for colour development and determination of liberated inorganic phosphate (Pi). The absorbance was measured using a Perkin-Elmer Lambda 25 ultraviolet–visible spectrophotometer at 660 nm.[12] One unit of enzyme activity (IU) was defined as the amount of enzyme that released 1 μg of inorganic phosphate per min under the assay conditions.

### Anionic surfactant

**Figure 1.**
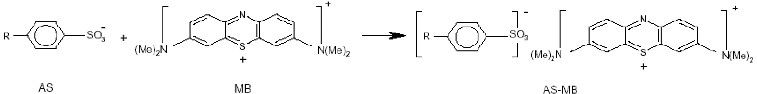
Mechanism of formation of ionic pair methylene blue–active substance (AS–MB) between the anionic surfactant (AS) and methylene blue (MB).

Anionic surfactant was determined by a spectrophotometric method using methylene blue. The method for determining the concentration of methylene blue-active substance (MBAS) in the detergents was that adapted from *Standard Methods for the Examination of Water and Wastewater* [13] and it was described by Ojo and Oso.[14] The method is based on the following reaction (Figure 1).

The percentage degradation was then calculated:(1) 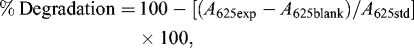
where *A*_625exp_ is the absorbance of a test sample, *A*_625blank_ is the absorbance of a blank sample and *A*_625std_ is the absorbance of a standard sample at 625 nm.

### Concentration of total and free organic acids

The concentration of free and total organic acids was determined by ion exchange chromatography according to the method of Bullen et al., [15] as previously described in greater detail.[16] The results were presented as percentages.

### Total dry biomass

The mycelia previously removed from the fermentation broth were washed with sterile distilled deionized water several times. Both filter paper and mycelia were then dried in an oven at 80 °C to a constant weight. The dry weight of the mycelia was determined by subtracting the initial weight of the filter paper from the weight of mycelia and filter paper.

### Statistical analysis

All experiments were performed in triplicate and results were expressed as means ± standard deviation. For statistical analysis, the following tests were used: Mann–Whitney, Kruskal–Wallis and the test for correlation coefficient by SPSS (Chicago, IL) statistical software package (SPSS for Windows, v. 13, 2004). Coefficient of correlation was tested at 0.05 and 0.01 level of significance.

## Results and discussion

The influence of detergent at 0.3% and 0.5% concentration on the changes of pH, redox potential, the concentration of organic acids, enzyme activity and total dry weight biomass of *P. chrysogenum* were examined. At the same time, the fungal ability to degrade the commercial detergent and utilize the degradation products as carbon and energy sources was tested. These parameters were monitored during the different physiological growth phases. The tested concentrations of detergent are several thousand-fold higher than those allowable and lethal for most living beings.

### Fungal growth

**Figure 2.**
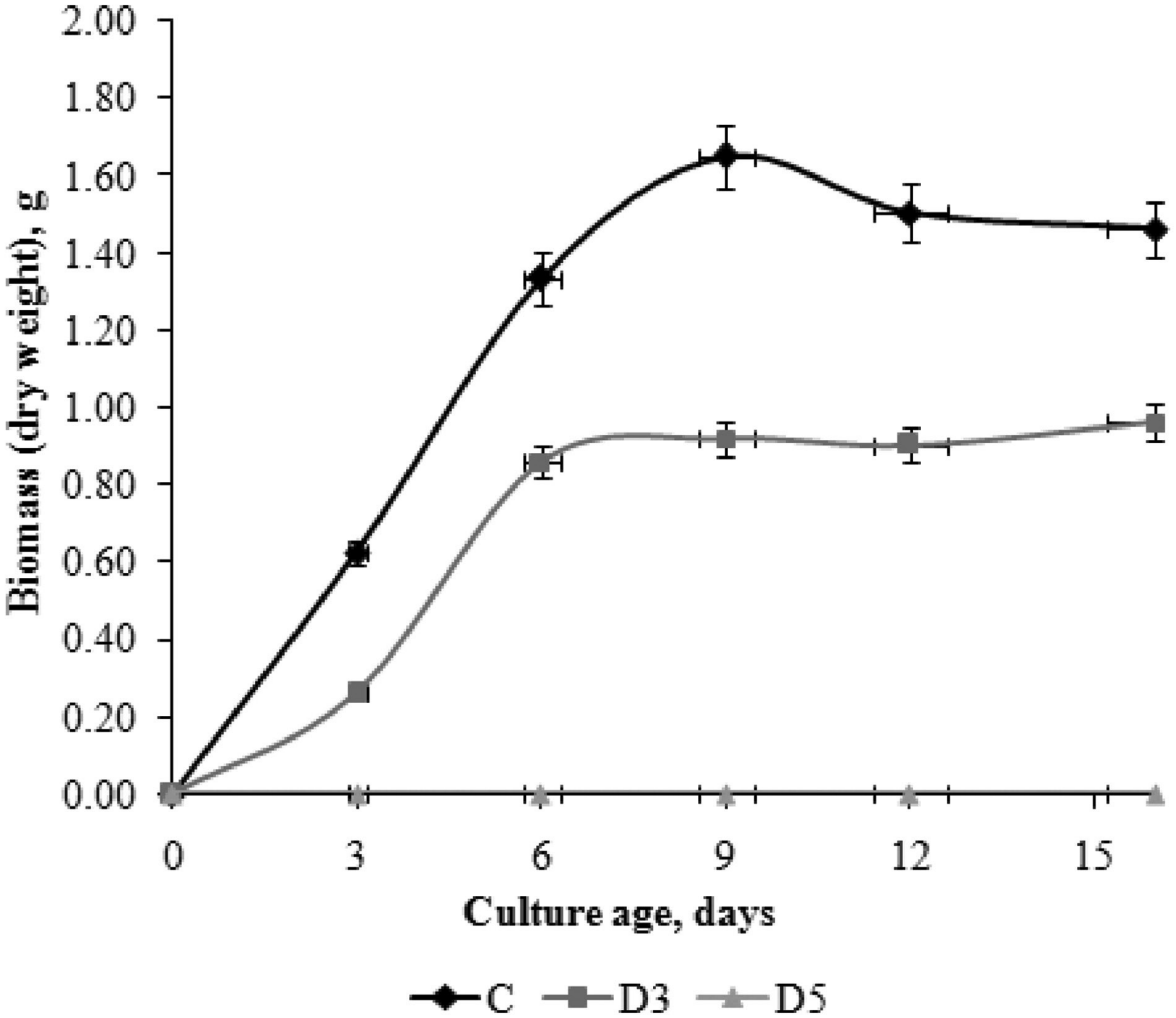
Growth curve based on total dry weight biomass (g) of *P. chrysogenum* cultivated in Czapek–Dox medium without detergent (C), with 0.3% detergent (D3) and 0.5% detergent (D5).

As generally known, chemical composition of the growth media and the experimental conditions influence the development of fungi and total biomass. Many investigations show that Czapek–Dox liquid medium has good properties for fungal cultivation and high biomass production.[17] Our results (Figure 2) showed that the studied *P. chrysogenum* isolate cultivated in Czapek–Dox liquid medium (C) had monophasic exponential growth from inoculation until day 6. At the end of the exponential and beginning of the stationary phase (day 6–9), the maximal total dry weight biomass 1.6423 g was observed. On day 12, autolysis began due to exhausting of sucrose from the medium, which was reflected as an insignificant reduction of biomass at the end of the experiment. These results are in accordance with many others.[18,19] The presence of different pollutants in the growth medium affects fungal growth and development. The ability of a surfactant to exert a fungicidal effect largely depends on the microbial species, the size of the hydrophobic portion of the surfactant molecule and the applied concentration.[14] The detergent at 0.5% showed a fungicidal effect and biomass was not detected in medium D5 during the experimental period. Interestingly, the fungus cultivated in D3 medium (0.3% detergent concentration) showed monophasic exponential growth, but the stationary phase was prolonged to day 16, probably due to utilization of detergent degradation products as an additional carbon source. The maximal total dry weight biomass in D3 medium was measured on day 16, but it was 61.84% less than in C medium. The reduction of total dry weight biomass can be explained by toxic effects of some degradation products or the detergent itself on fungal growth. As it is well known, detergents have an inhibitory effect on the enzymes involved in key metabolic pathways. Fungal growth is also influenced by physico-chemical interactions between surfactants and fungal structures such as membranes and cell walls. Many investigations confirm that the anionic surfactant sodium dodecyl sulphate (SDS) inhibits growth, whereas non-ionic surfactants such as Triton X-100 and Tween 80 are well tolerated by fungi.[20] In the presence of the anionic detergent tested in this study, the fungal isolate showed ability to grow rapidly and fungal biomass was noted on day 3. This result indicated a very short adaptation period, which suggests that the fungus is genetically predisposed to the tested concentrations of detergent and synthesizes the enzymes necessary for degradation. The highest percentage of biodegradation of anionic detergent component was measured during fungal exponential growth. This is in agreement with other authors who confirmed that the degradation rate of surfactant is concomitant with cellular growth.[21,22]

### Detergent biodegradation rate

**Figure 3.**
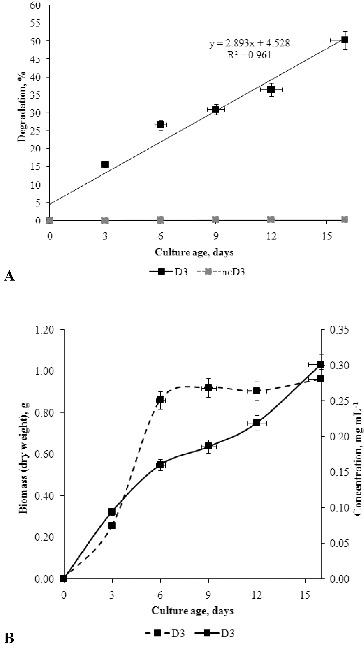
Percentage of biodegradation of detergent in the fermentation broth of *P. chrysogenum* grown in D3 medium (with 0.3% detergent) compared with negative non-inoculated control (ncD3) (A). Relationship between concentration of decomposed anionic surfactant (solid line) and dry weight biomass (dotted line) (B).

In the next step of our study, the biodegradation of commercial detergent by *P. chrysogenum* was evaluated only in inoculated liquid media supplemented with detergent at a concentration of 0.3% (D3 medium). Because the detergent showed fungicide effect at concentration 0.5%, further measurements were not carried out in medium D5. Non-inoculated liquid media with addition of detergent at 0.3% was used as a negative control. The negative control showed that the tested detergent was chemically stable in the experimental conditions (Figure 3(a)). The initial concentration of detergent of 3 mg mL^−1^ (in D3 medium) decreased continuously with the mycelial growth. During the first three days, the fungus decomposed 15.63% of the initial detergent concentration. From day 3 to day 6 the biodegradation percentage was 26.73%; from day 6 to day 9 it was 31.03%; from day 9 to day 12, 36.567%; and on day 16 (at the end of the experiment), the detergent concentration was 1.509 mg mL^−1^, which it was total 50.2% of the initial detergent concentration. These results show that the fungus decomposed the highest percentage of detergent during the exponential growth phase. In the stationary growth phase, the fungus degraded a lesser amount of the anionic component of the detergent.

The tested detergent contains 20% anionic surfactant, which was confirmed by MBAS assay. That is, the initial concentration of anionic surfactant in D3 medium was 600 μg mL^−1^. As shown in Figure 3(b), the fungus decomposed about 301 μg mL^−1^ of anionic surfactant in medium D3 in 16 days. It is well known that the biodegradation rate depends on the type and concentration of surfactants and on the microorganism species. According to Amirmozafari et al.,[23] the two bacterial strains *Pseudomonas betelli* MH1 and *Acinetobacter johnsoni* MH2 have the ability to decompose 93.6% and 84.6% of the initial concentration of linear alkyl benzene sulphonates (LAS) at alkaline pH and room temperature in five days. The highest degradation of LAS occurs during the logarithmic growth phase.[23] The results of Garon et al. [20] showed that *P. chrysogenum* tolerates non-ionic surfactants, such as Tween 80 (0.324 mmol L^−1^), well and in the presence of this surfactant can enhance the biodegradation of some other pollutants such as fluorene from 28% to 61% after two days. Jerabkova et al. [24] demonstrated that *Pseudomonas* cultures in continuous bioreactors decrease about 70% of anionic surfactant levels after 20 days. However, Schleheck et al. [25] revealed that *Citrobacter* spp. have the ability to degrade over 90% of anionic surfactant after 35 h of growth. Hosseini et al. [26] showed that *Acinetobacter johnsoni* strains can utilize 94% of the original SDS levels after five days. Our results provided direct evidence that *P. chrysogenum* actively uses commercial detergent of the anionic type added to liquid Czapek–Dox nutrient medium for its metabolism. It is important to note that although the degradation percentage observed by us is lower than that achieved with bacterial strains, the concentrations tested in this study were higher. Moreover, the above-mentioned reports on biodegradation by bacterial strains use pure anionic components, whereas the commercial detergent used in our study is very complex and closer to the ones found in real life.

### pH and redox potential

**Figure 4.**
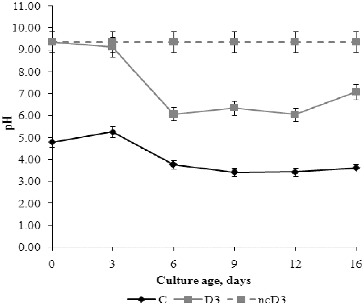
pH values of fermentation broth during *P. chrysogenum* growth in Czapek–Dox medium with (D3) and without (C) 0.3% detergent, compared to non-inoculated control (ncD3).

The changes in pH and redox potential were evaluated because these parameters are very important for regular fungal growth and development and have application in the monitoring of bioremediation processes. The optimum external pH for fungal growth is between 4.5 and 5 in the acidic range, while pH above 5 is necessary for spore germination. Figure 4 shows the changes in pH in the fermentation broth during the growth of the fungus. Before inoculation, the initial pH values were 4.80 and 9.35 in C and D3 medium, respectively. A negative non-inoculated control with detergent (ncD3) was tested in parallel and no changes in its pH value were observed during the experimental period. There were changes in the pH values of C and D3 media in relation to their composition and the growth phases of the fungus. These changes are influenced by the uptake of anions or cations from the medium by the fungal cells.[27,28,29] The pH value of C medium increased slightly from inoculation until day 3, and then decreased during the late exponential growth and stationary phase. From the beginning of autolysis until the end of the experiment, pH value increased insignificantly. These changes in the pH value of C medium are an additional indicator of cell autolysis during which some alkaline products are released, slightly altering the pH. The pH value of the D3 medium decreased during the exponential growth phase from inoculation until day 6. In the stationary phase, the pH values reached a plateau; and at the end of the experimental period the pH value increased towards the neutral range. The tested detergent behaved as a strong alkaline agent, which changed the pH of the medium from 4.5 to 5.0 units. Decreasing of media pH is observed during extensive mycelium development of many fungal species such as *Glomus intraradices*,[30] *Fusarium oxysporum*,[31] etc. The results from our study could suggest that fungi have different mechanisms for regulation of environmental pH which depend on the initial pH. It could be speculated that the presence of detergent in the medium probably induces the expression of different sets of genes, compared to control, as reflected in the regulation of external pH.

**Figure 5.**
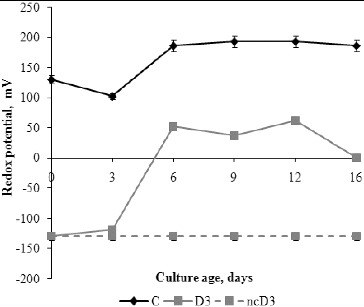
Redox potential values of fermentation broth during *P. chrysogenum* growth in Czapek–Dox medium with (D3) and without (C) 0.3% detergent, compared to non-inoculated control (ncD3).

The initial redox potential values, before inoculation were 130 mV and –130 mV in C and D3 media, respectively.[Figure 5] A negative non-inoculated control with detergent was also tested and during the experimental period no changes in its redox potential value were observed. The redox potential values of C and D3 media changed in relation to the media composition and fungal growth phases. The highest changes of redox potential of C medium were measured from day 3 to day 6, which corresponds to the exponential growth phase. During the stationary phase and autolysis phase there were no significant changes in the redox potential. The redox potential of D3 medium increased until day 6, whereas during the stationary phase the changes in the redox potential were insignificant. A decrease in the redox potential was observed on day 16, when its value was almost zero.

Many investigations demonstrate the influence of the redox potential value on the activity of some enzymes such as ADP-glucose pyrophosphorylase,[32,33] the activity and binding of α-glucan, water dikinase (SEX1) to starch granules [34] and possibly the activity of β-amylase.[35] The positive value of the redox potential in C medium is due to the presence of sucrose and mineral salts, whereas addition of detergent in the growth medium causes anaerobic conditions and negative values of the redox potential. Sucrose in the growth medium is hydrolysed by the catalytic activity of invertase to reducing sugars (glucose and fructose). Glucose is transported into the cell by the phosphotransferase system and is catabolized to pyruvate whose further fate depends on the availability of oxygen. With an electron acceptor, the nicotinamide adenine dinucleotide (NADH) produced in glycolysis and the citric-acid cycle can be oxidized by the electron transport chain to produce energy by oxidative phosphorylation.[36] Probably, electron acceptors are sulphates from the anionic surfactant in the medium with detergent, since the redox potential was increased in these media. During the exponential growth, the changes in the redox potential were most pronounced and followed the opposite trend to those of the pH curves (*r* = –0.994, *P* < 0.01) and the percentage of detergent degradation. These results indicate a role of H^+^-ATPase in the regulation of environmental pH.

### Production of free and total organic acids

**Figure 6.**
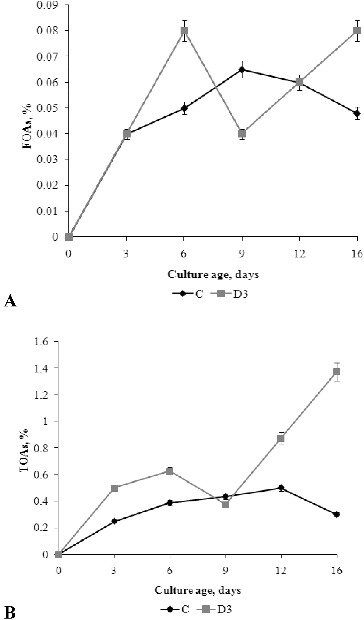
Concentration of FOAs (A) and TOAs (B) in the fermentation broth of *P. chrysogenum* grown in Czapek–Dox medium with (D3) and without (C) 0.3% detergent, compared to non-inoculated control (ncD3).

Since organic acids play a key role for alkali tolerance, especially for the intracellular ionic homeostasis, we measured the concentrations of free organic acids (FOAs) and total organic acids (TOAs) in the fermentation broth during fungal growth.[Figure 6] FOAs concentration increased in C medium during the exponential growth phase from 0.04% to 0.065%, which was the highest concentration measured in this medium. After day 9, the concentration of FOAs decreased gradually until the end of the experiment. In D3 medium, the highest amount of FOAs was produced (0.08%) on day 6 and day 16 and these values were somewhat higher than the maximal value in C medium. Decreasing of FOA concentration was measured in D3 medium in early and late stationary phase but these changes were not statistically significant.

The concentration of TOAs in the control medium was highest on day 12; and with aging of the culture (day 16), the TOAs concentration decreased slightly. On the contrary, in the medium supplemented with detergent (D3), the TOAs concentration increased with aging of mycelia (day 12–16) and on day 16, was about four-fold higher than in the control (C) medium.

According to Andersen et al.,[37] organic acids can be transported across the plasma membrane into the medium in the form of organic acid anions or fully protonated. The high percentage of fully protonated forms strongly contributes to medium acidification, while organic acid anions would contribute only a little. Accumulation of OAs might be result of the deficit of negative charge, and OAs metabolic regulation might be a key pathway for keeping ionic balance and pH stability. Under alkali stress, the content of inorganic anions decreases, which causes considerable accumulation of OAs, especially malate and citrate, indicating that OAs secretion may be important in the adjustment of external pH.[38,39] When sucrose is the sole carbon source, acetic acid is the predominant organic acid in the FOA pool.[40] In consideration to our results, it was expected that the large pH differences between the experimental media would be related to significantly different amounts of FOAs. The statistical analysis, however, showed no significant differences of organic acid amount between C and D3 media, suggesting that the type and strength of organic acids, rather than their amount, influenced the external pH. It is generally known that organic acids can serve another purpose, such as charge balance, energy spilling, chelation of trace elements and biomass build-up.

### Activity of acid and alkaline invertase in media

**Figure 7.**
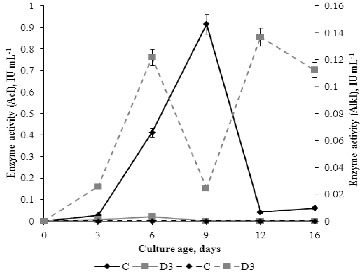
Profile of extracellular acid invertase (AcI) activity (solid line) and alkaline invertase (AlkI) activity (dotted line) of *P. chrysogenum* cultivated in Czapek–Dox medium with (D3) and without (C) 0.3% detergent.

The enzymatic hydrolysis of sucrose to glucose and fructose is catalysed by invertase. According to Poonawalla et al.,[41] *P. chrysogenum* has both extracellular and intracellular invertase with different subcellular localization (cell wall, vacuole or cytosol), solubility (soluble or insoluble in low ionic strength buffer), optimum pH (acid or neutral/alkaline) and isoelectric point (pI).[42] In our study, the alkaline and acid invertase activities of *P. chrysogenum* were measured in the fermentation broth during fungal development. The obtained results (Figure 7) confirmed that the activity of these enzymes depends on the type of media and the stage of fungal development. No alkaline invertase (AlkI) activity was detected in C medium, whereas variable AlkI activity was expressed in D3 medium. Acid invertase activity (AcI) was expressed in C medium throughout the whole experimental period, and the maximum enzyme activity was measured on day 9. The activity of this enzyme was extremely weak in medium D3 and was completely inhibited after day 6. There was correlation between the redox potential and the activity of acid invertase (*r* = 0.807) and redox potential and alkaline invertase activity (*r* = –0.772).

These results indicate that different sugar transport systems operate in C and D3 media and two different invertases function in D3 medium. AcI activity corresponded with the concentration of FOAs in C medium, which is in agreement with the fact that organic acids originate from the oxidative pathways of hexoses. Correlation between FOA concentration of and AlkI activity was not observed in C medium. Although the AcI activity was low, the concentration of FOAs and TOAs was higher in D3 medium as compared to the control. These differences in the concentration of organic acids between the media could be a result of different initial pH value as well as of the presence of detergent degradation products such as some fatty acids, sulphophenyl carbonic acids, etc.

### Activity of alkaline phosphatase in media

**Figure 8.**
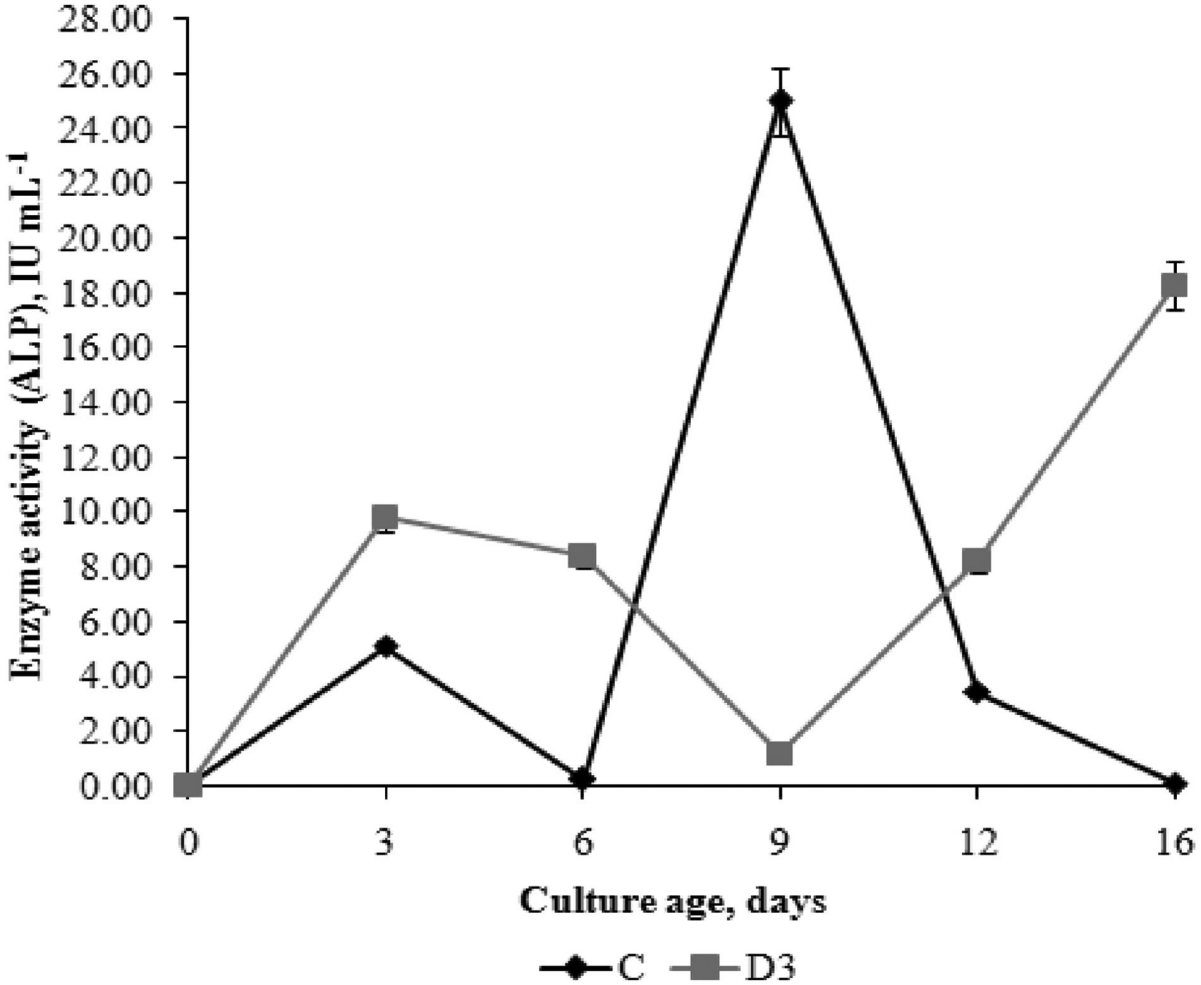
Profile of extracellular alkaline (ALP) phosphatase activity of *P. chrysogenum* in Czapek–Dox medium with (D3) and without (C) 0.3% detergent.

ALPs hydrolyse the esters and anhydrides of phosphoric acid, etc.[43] ALP enzymes are involved in various biological processes, e.g. cell cycle, differentiation and others. Although the optimal pH value for enzyme activity is around 9, many studies show that ALP is active in a wide pH range, from 3 to 11, which was also confirmed in our experiments.[Figure 8] The ALP activity in C medium was highest on day 9, when the fungal biomass and AcI activity were also maximal. In D3 medium, the highest ALP activity was measured on day 16, when the highest percentage of detergent biodegradation was observed in this medium. The maximal ALP activity was significantly higher (*P* < 0.05) in C medium than in the medium with detergent. This was expected, considering the specific effect of the enzyme on β-glycerophosphate, the composition of the growth medium and the invertase activity. ALP also hydrolyses more easily monoesters originating from the carbohydrate metabolism than ester bonds in the alkyl chain of surfactants.[44] According to Koffi et al.,[45] SDS displays a strong inhibitory effect (about 98%) on phosphatase activity. The results from our study showed that ALP activity was inhibited by detergent (about 24%) as compared to the control.

## Conclusions

The results from this study showed that commercial detergent added to a liquid Czapek–Dox nutrient medium acts as a stress and nutrient factor on the metabolism of *P. chrysogenum* at a concentration of 0.3%, whereas at a 0.5% concentration it had a fungicidal effect. The stressful effect of the detergent was manifested in alkalization of the substrate, reduction of the oxidation–reduction potential, inhibition of invertase and phosphatase activity and reduction of total dry weight biomass. These changes were most pronounced at the stage of spore germination and early stages of mycelial development. Some organic acids produced by the fungus in the presence of detergent could have practical applications in biotechnology and pharmacology. Which is more, the results suggest that *P. chrysogenum* could degrade anionic detergent components and utilize the degradation products for its metabolism. This ability could be of practical interest in wastewater treatment plants and non-invasive and cost-effective bioremediation technologies. 
